# The effect of immediate postoperative intravenous administration of ferric carboxymaltose after autologous free-flap breast reconstruction

**DOI:** 10.1038/s41598-022-23976-2

**Published:** 2022-11-09

**Authors:** Joseph Kyu-hyung Park, Seungjun Lee, Chan Yeong Heo, Jae Hoon Jeong, Yujin Myung

**Affiliations:** Department of Plastic and Reconstructive Surgery, Seoul National University College of Medicine, Seoul National University Bundang Hospital, 300 Gumi-Dong, Bundang-Gu, Seongnam-Si, 463-707 Gyeonggi-Do Korea

**Keywords:** Drug therapy, Health care, Surgery, Surgical oncology

## Abstract

Intravenous ferric carboxymaltose (IV-FCM) can effectively correct perioperative anemia in patients undergoing major surgeries. However, its efficacy and side effects in patients undergoing free flap-based breast reconstruction are yet to be investigated. At our institution, from year 2020, patients with breast cancer undergoing abdominal free flap-based breast reconstruction were injected 500 mg of IV-FCM immediately post-operation. Propensity-matched 82 IV-FCM injected (study group) and 164 historical control group patients were retrospectively analyzed for transfusion rates, changes in hematological parameters, and flap or donor-site related complications. The major and minor complication rates related to the operation site were similar between the two groups. There was no significant difference in the transfusion rate between the two groups (control 29.9% vs. study 32.9%, p = 0.71). However, the total amount of transfusion required was significantly higher in the historical control group (control—53.2% 1 pack, 42.6% 2 packs, 4.3% 3 packs of RBC vs. Study—66.7% 1 pack, 33.3% 2 packs, p = 0.02) than in the study group. Additionally, the historical control group showed a significantly higher drop in red blood cell count, hemoglobin, and hematocrit levels from postoperative days 1–2 and 2–3 compared to the study group. Immediate postoperative use of IV-FCM in free flap-based breast reconstruction was well tolerated by patients and reduced overall transfusion volume.

## Introduction

Free flap-based autologous breast reconstruction is a widely used reconstruction method. Its widespread adoption could be attributed to several benefits, including the availability of sufficient soft tissue, great cosmetic outcome, outstanding long-term patient satisfaction, and minimal donor site morbidities^1,2^. However, its limitations include a long operation time, a larger operating field, and a relatively higher estimated blood loss (EBL)^3,4^. With a reported average EBL of 1300 mL, higher EBL during free flap-based breast reconstruction leads to a higher risk of perioperative anemia than other reconstructive methods. A recent study showed that approximately 9–19% of free flap-based breast reconstruction recipients received postoperative blood transfusion^3,5^. In addition to the higher EBL associated with free flap-based breast reconstruction, other factors such as chemotherapy and radiotherapy increase the risk of developing anemia in patients with breast cancer.

Allogenic or autologous red blood cell (RBC) transfusion is the only treatment option in patients with severe postoperative anemia. However, blood transfusion is an invasive procedure associated with risks of hematogenic infection, hyperemia, hypersensitivity, and possibly prolonged hospital stays^6,7^. Some studies have also indicated the possibility of increased flap-related complications in patients with free flap-based breast reconstruction receiving intraoperative or postoperative transfusions^8–10^. The costs of packed allogenic RBCs are high; however, the blood donation rate has decreased significantly in many parts of the world since the coronavirus disease 2019 (COVID-19) pandemic^11,12^.

Previous studies have illustrated the efficacy of intravenous ferric carboxymaltose (IV-FCM) in correcting iron deficiency anemia in patients with chronic anemia^13,14^ as well as patients undergoing abdominal surgery^15^, hip surgeries^16^, and even childbirth^17^. Especially during major surgeries with high EBL, perioperative injection of IV-FCM has been found to reduce transfusion rates while improving postoperative hematological parameters (hemoglobin [Hb], ferritin, and hematocrit [Hct] levels)^14^. The number of institutions utilizing intravenous therapy such as ferric carboxymaltose, iron sucrose, sodium ferric gluconate, and ferrumoxytol for patient blood management (PBM) protocols has increased rapidly.

However, to the best of our knowledge, no study has examined RBC transfusion-sparing benefits of IV-FCM in patients undergoing free flap-based breast reconstructions. Since the year 2020, our institution has adopted a new postoperative protocol involving immediate postoperative injection of a single dose of IV-FCM (500 mg). In this study, we examined the benefits of IV-FCM in patients with breast cancer undergoing breast reconstruction using free muscle-sparing transverse rectus abdominis muscle (MS-TRAM) flap by comparing transfusion rates, RBC counts, Hb levels, and hematocrit levels between patients receiving IV-FCM against a historical control group.

## Results

**Figure 1:**
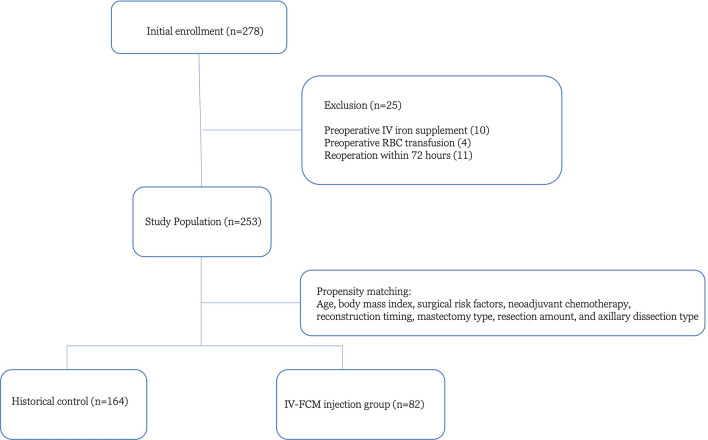
Flowchart illustrating the exclusion and inclusion criteria of the present study.

A total of 278 breast cancer patients who underwent free MS-TRAM flap-based breast reconstruction were included; 84 patients received immediate postoperative IV-FCM injections, while 194 patients did not. Fourteen patients who received preoperative transfusion or iron replacement therapy due to severe anemia and 11 patients who underwent re-operation within 72 h of the initial flap operation were excluded, leaving a total of 253 patients for study enrollment. Of these patients, propensity score matching based on age, BMI, underlying diseases, and operative details resulted in a total of 246 patients for further analysis (c-statistic of 0.803). Eighty-two propensity-matched IV-FCM injected patients (study group) and 164 historical control group patients were analyzed (Fig. 1).

Propensity-matched groups did not show a significant difference in patient demographics [age, body mass index (BMI), surgical risk factors, and neoadjuvant chemotherapy]. In addition, there was no difference in the reconstruction timing, mastectomy type, resected tissue volume, and axillary dissection type between the two groups (Table 1 Patient demographics).

**Table 1:** Demographics.

		Historical control group	Treatment group (IV ferinject)	p-value
Patients (n)		164	82	
Age		47.7 ± 10.3	48.1 ± 11.2	0.56
BMI		25.1 ± 4.8	24.8 ± 4.3	0.37
Hypertension		9	6	0.21
DM		4	3	0.16
Reconstruction timing	Immediate	119	63	0.49
	Delayed	12	9	
Neoadjuvant chemotherapy	Yes	33	21	0.53
	No	97	52	
Mastectomy	TM	15	9	0.46
	SSM	50	27	
	NSM	96	31	
Resection amount		299.1 ± 36.8	308.4 ± 40.1	0.34
Axillary dissection	ALND	50	28	0.27
	SLND	111	64	
Mild complication (no reoperation)	Hematoma	5	2	
	wound infection	3	3	
	donor site bulging / hernia	1	1	
Ferinject side effects	Hypersensitivity	N/A	1	
	Skin discoloration		1	
	others		0	

There were two cases of IV-FCM-related side effects. There was one case of skin rash and irritation after IV-FCM injection, which subsided after medication, and one case of mild skin discoloration, which resolved spontaneously within 2 weeks. In other patients, IV-FCM was well tolerated.

The rates of major and minor complications related to the operation site (the flap or the donor site) were similar between the study and the control groups. In the historical control group, there were two cases of re-admission (1.2%), one case of re-operation (0.6%), five cases of hematomas (3.0%), 16 cases of delayed wound healing (9.7%), three cases of wound infections (1.8%), one case of donor site bulging (0.6%), and no cases of fat necrosis (0.0%). In the study group, there were zero cases of re-admission (0.0%), one case of re-operation (1.2%), two cases of hematomas (2.4%), 10 cases of delayed wound healing (12.2%), three cases of wound infections (3.6%), one case of donor site bulging (1.2%), and zero cases of fat necrosis (0.0%).

**Figure 2:**
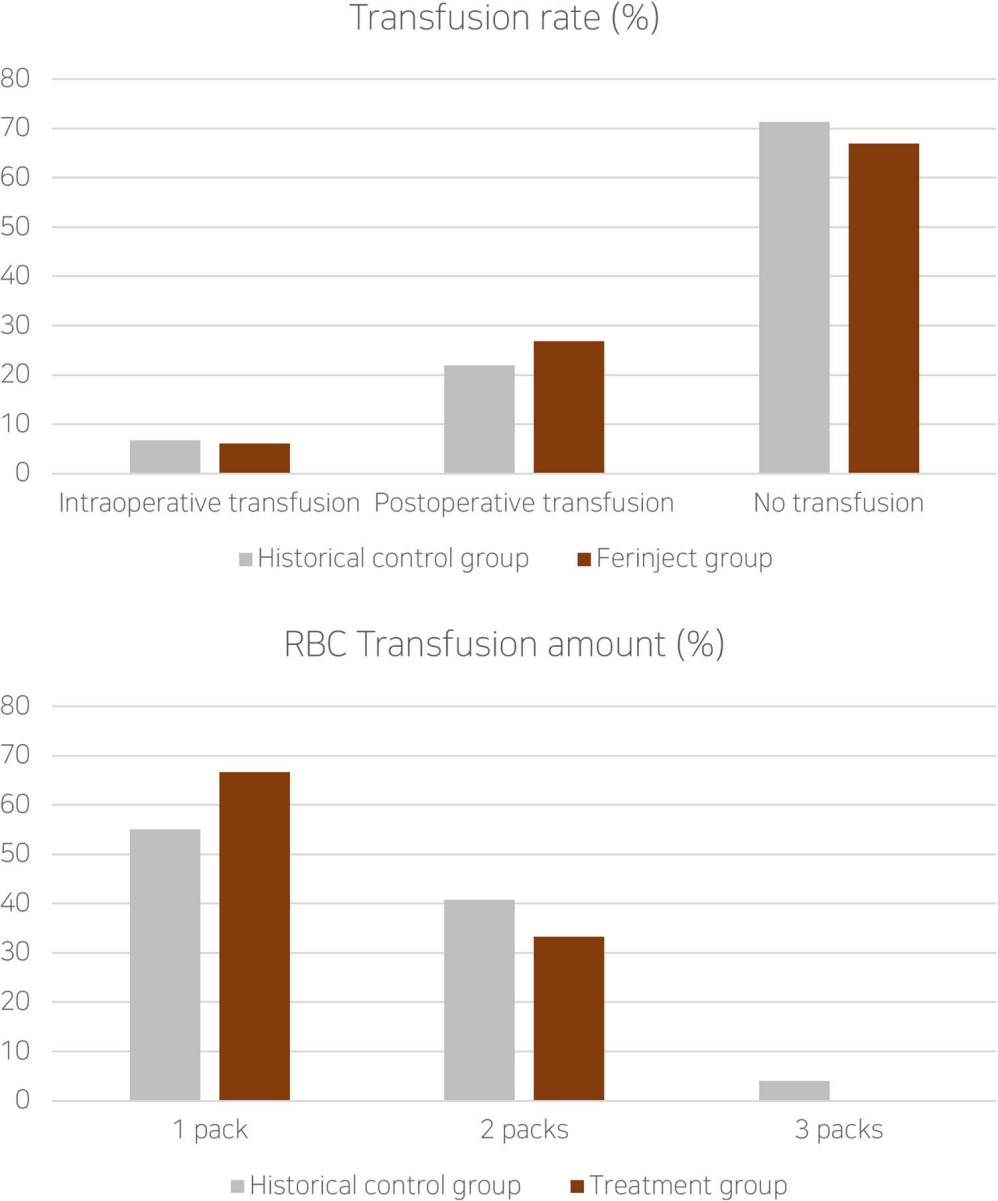
Transfusion rate (%) between control and IV-FCM group. There was no significant difference (above). Amount of transfusion (red blood cell [RBC] packs) in control and IV-FCM group. The difference was significant (below).

Preoperative hemoglobin and hematocrit levels were not significantly different between the historical control and the study groups (control—Hb 12.50 g/dL, Hct 37.7% vs. Study—Hb 12.44 g/dL, Hct 37.4%, *p*-value = 0.85). In the historical control group, 47 out of 164 patients (29.9%) received blood transfusions during their hospitalization. Eleven patients (23.4%) received blood transfusions intraoperatively, while 36 patients (76.6%) received them postoperatively due to low Hb and Hct levels. Among the 47 patients receiving transfusions, 25 (53.2%) received one pack, 20 (42.6%) received two packs, and two patients (4.3%) received three packs of RBC. In the study group, 27 out of 82 patients (32.9%) received blood transfusions. Five patients (18.5%) received transfusions intraoperatively, while 22 (81.5%) received them postoperatively. Of these patients, 18 (66.7%) received one pack and 9 (33.3%) received two packs of RBC. There was no significant difference in the transfusion rate between the two groups (Fig. 2, *p*-value = 0.71). However, the total amount of transfusion was significantly higher in the historical control group than in the study group (Fig. 2, *p*-value = 0.02).

**Figure 3:**
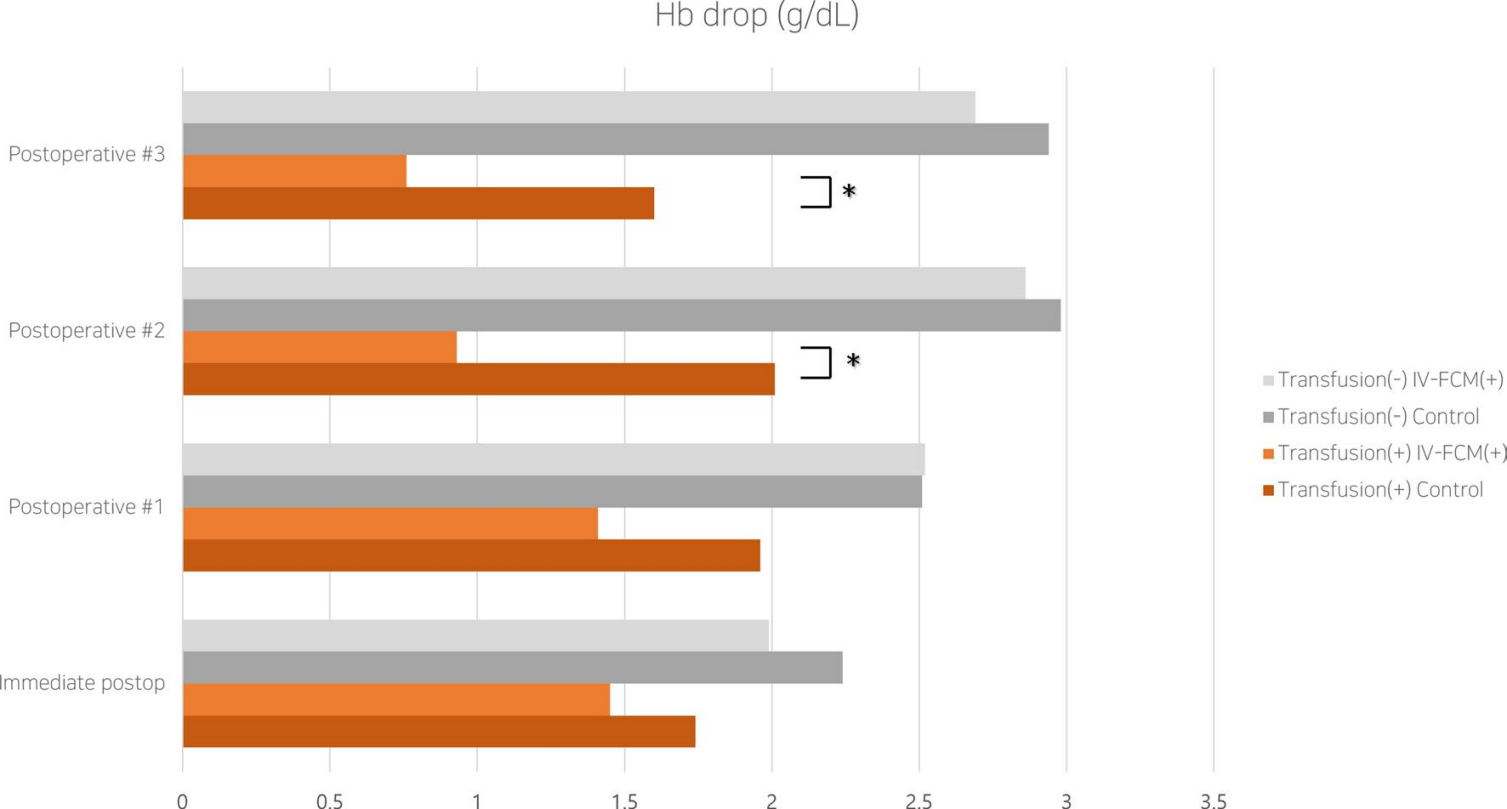
Change of hemoglobin levels illustrated graphically. The difference between the IV-FCM and the control groups in terms of RBC transfusion was significant on postoperative days #2 and #3 (asterisk).

**Figure 4:**
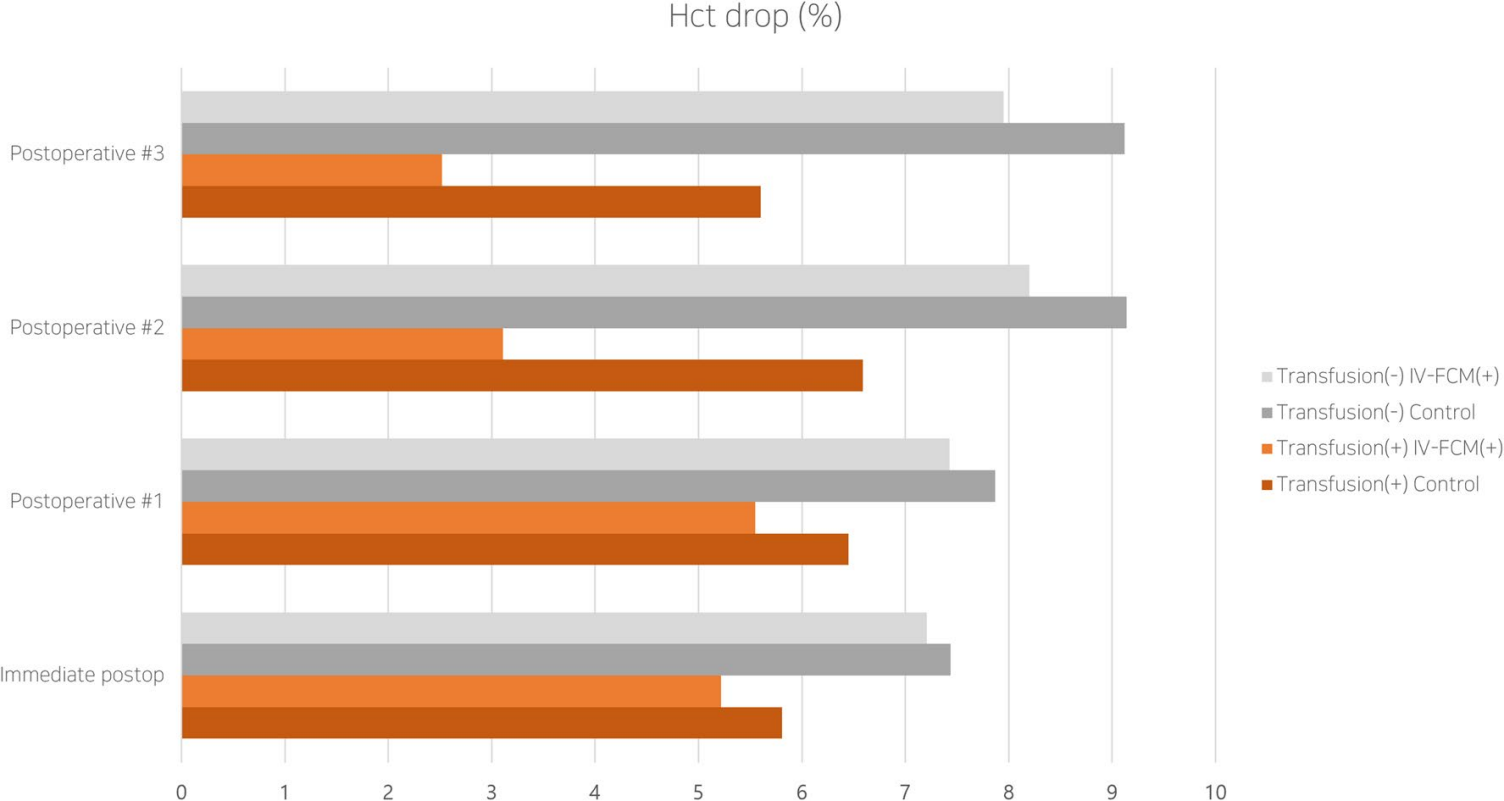
Change of hematocrit percentages illustrated graphically. The difference between the IV-FCM and control groups in terms of RBC transfusion was significant on postoperative days #2 and #3 (asterisk).

Subgroup analyses were performed to investigate the effect of IV-FCM in transfusion (+) and transfusion (−) groups. Within each group, hematological parameters and their postoperative changes were compared. Within the transfusion (+) group, 27 patients with IV-FCM treatment and 47 historical control patients were compared, while 55 patients with IV-FCM treatment and 117 historical control patients were compared within the transfusion (−) group. Within the transfusion (+) group, there was no significant difference in RBC count, Hb level, and Hct level drops immediately post-operation to postoperative day (POD) #1 between the study and control groups (Table 2). However, there was a significant drop in the RBC count, Hb level, and Hct levels from POD#1 to POD#2 as well as from POD#2 to POD#3 between the study and control groups. In contrast, such differences were not evident in the transfusion (−) group (Fig. 3, 4).

**Table 2:** Comparison of hemodynamic changes between the transfusion group and non-transfusion group from postoperatively.

		Transfusion (+)			Transfusion (−)		
		Control	IV-FCM(+)	p value	Control	IV-FCM (+)	p value
Patients (n)		47	27		117	55	
RBC drop (95% CI) (10^6/μL)	Immediate postop	0.62 (0.53,0.71)	0.48 (0.34,0.62)	0.13	0.73 (0.48,0.98)	0.65 (0.54,0.76)	0.48
	Postoperative #1	0.69 (0.57,0.81)	0.71 (0.54,0.87)	0.89	0.87 (0.82,0.92)	0.75 (0.65,0.85)	0.28
	Postoperative #2	0.75 (0.63,0.87)	0.5 (0.40,0.599)	0.04*	0.9 (0.84,0.96)	0.99 (0.94,1.04)	0.37
	Postoperative #3	0.62 (0.55,0.69)	0.3 (0.17,0.43)	0.01*	0.95 (0.91,0.99)	0.88 (0.83,0.93)	0.56
Hb drop (95% CI) (g/dL)	Immediate postop	1.74 (1.49,1.98)	1.45 (1.06,1.84)	0.25	2.24 (1.73,2.75)	1.99 (1.70,2.28)	0.22
	Postoperative #1	1.96 (1.59,2.33)	1.41 (0.86,1.96)	0.05	2.51 (2.36,2.66)	2.52 (2.32,2.72)	0.94
	Postoperative #2	2.01 (1.71,2.31)	0.93 (0.58,1.38)	< 0.01*	2.98 (2.88,3.08)	2.86 (2.72,3.00)	0.55
	Postoperative #3	1.6 (1.37,1.83)	0.76 (0.42,1.1)	0.02*	2.94 (2.80,3.08)	2.69 (2.53,2.85)	0.29
Hct drop (95% CI) (%)	Immediate postop	5.81 (5.08,6.53)	5.22 (3.97,6.47)	0.41	7.44 (6.48,8.40)	7.21 (6.33,8.09)	0.4
	Postoperative #1	6.45 (5.48,7.42)	5.55 (4.07,7.03)	0.44	7.87 (7.39,8.35)	7.43 (6.82,8.04)	0.46
	Postoperative #2	6.59 (5.62,7.55)	3.11 (1.96,4.26)	< 0.01*	9.14 (8.72,9.56)	8.2 (7.65,8.75)	0.09
	Postoperative #3	5.6 (4.89,6.31)	2.52 (1.53,3.50)	< 0.01*	9.12 (8.40,9.84)	7.95 (7.43,8.47)	0.07

Abbreviations : IV-FCM : Intravenous ferric carboxymaltose , RBC : Red blood cell count, Hb : Hemoglobin, Hct : Hematocrit.

## Discussion

To our best knowledge, our study is the first to examine the effects of IV-FCM in patients undergoing free flap-based breast reconstructions and the first to report the safety and efficacy of IV-FCM in reducing the amount of blood transfusion. Our results show that immediate postoperative injection of 500 mg of IV-FCM does not reduce the transfusion rate but reduces the total amount of transfusion needed during the acute postoperative period.

FCM is a macromolecular ferric hydroxide carbohydrate complex that, following intravenous injection, increases the overall serum iron level, leading to an increase in Hb levels by increasing serum ferritin and transferrin saturation levels^18^. Previous clinical studies have reported increased Hb levels 2–6 weeks after a single dose of IV-FCM^19^.

In a recent study based on patients undergoing cardiac surgery, combined therapy using IV-FCM and subcutaneous erythropoietin alpha decreased RBC transfusion rate during the acute postoperative period^20^. Furthermore, in a large-sized randomized clinical trial on patients undergoing gastrectomy, preoperative injection of a single dose of IV-FCM showed a significant increase in the serum Hb level as well as ferritin and transferrin saturation levels after 3 and 12 weeks post-operation, respectively^15^.

Unlike our study's protocol, most previous studies on IV-FCM performed preoperative injection instead of a postoperative injection of IV-FCM. These findings suggest that IV-FCM would be more beneficial if the injection is performed 1–4 weeks before the operation. However, due to the shortage of staff during the COVID-19 pandemic, outpatient-based injection of IV-FCM was only possible in severely anemic patients at our institution. While the safety of IV-FCM has been well established, 1–4 weeks preoperative IV-FCM injection was not possible due to the concern of possible side effects^21^. To circumvent these issues, we performed IV-FCM injection immediately after transferring the patient from the post-anesthesia care unit to the ward, where the nursing staff could monitor the patient, and the attending physicians could rapidly treat any adverse effect. Despite the latency in the Hb boosting effect of IV-FCM, our results showed a reduction in the total RBC transfusion quantity, which provides evidence-based support for using IV-FCM in patients undergoing free flap-based breast reconstruction.

Another study on orthopedic hip surgery patients showed reduced transfusion amounts without affecting the clinical outcomes with an immediate postoperative injection of IV-FCM^16^. In their protocol, IV-FCM was injected if the immediate postoperative Hb level was less than 10 g/dL or showed a drop of more than 3 g/dL compared to the preoperative value. RBC transfusion was performed if the Hb level was below 8 g/dL or the patients demonstrated symptoms of acute anemia. Apart from this study, published data on immediate postoperative injection of IV-FCM are scarce.

While RBC transfusion is currently the only treatment option for low postoperative Hb levels, it has numerous acute and chronic possible side effects. Consequently, many have advocated RBC transfusion as a last resort and have searched for other treatment options. Furthermore, some studies have shown increased flap-related and other postoperative complications in free flap-based breast reconstructed patients who received postoperative blood transfusions. Increased complication rates have been reported in head and neck reconstruction patients^9,22^ and breast cancer patients undergoing free flap reconstructions^3,5,23^.

The exact mechanism by which blood transfusion affects free flap-based breast reconstruction outcomes remains unknown. Some studies have proposed that increased levels of inflammatory cytokines, such as interleukins and tumor necrosis factor-alpha, can disrupt iron homeostasis^10^, and such disruption can cause thromboembolism^24^. In our study, the rate of flap or donor site-related complications was similar between the IV-FCM and the control groups. Therefore, the use of IV-FCM was considered to have reduced transfusion complications.

Furthermore, blood donation rates have declined in recent years, especially in developing countries^11,12^. The situation has worsened due to the recent COVID-19 pandemic, leading to the limited availability of packed RBC. Consequently, PBM has become increasingly important in clinical settings to reduce the demand for blood transfusions.

There are several limitations to this study. First, long-term laboratory results were not available for our historical control group, and the analysis of long-term results of IV-FCM was not possible. Second, while propensity matching was performed to eliminate biases caused by covariables between the two groups, the lack of randomization leaves room for possible bias caused by unpredicted confounding factors. Third, serum iron profile, which better reflects iron deficiency, were not available for analysis. Cancer patients, especially those who underwent neoadjuvant chemotherapy, could have anemia for causes other than iron deficiency. These factors were not considered in this study.

In conclusion, our study shows that immediate postoperative use of IV-FCM in free flap-based breast reconstruction is well tolerated by the patient. To the best of our knowledge, this is the first study to show the efficacy and safety of IV-FCM in patients undergoing free-flap based breast reconstruction. It helps reduce overall transfusion volume and promotes faster postoperative recovery of hematological parameters. These findings can be further confirmed by large-scale observational studies in the future.

## Methods

### Patients

This retrospective cohort study was conducted in accordance with the ethical standards of the Declaration of Helsinki. Informed consent was obtained from all patients and the study was approved by the Institutional Review Board of our institution (Seoul National University Budang Hospital Instituitional Review Board Approval No. B-2112–724-103). Patients with Breast cancer who underwent breast reconstruction using free MS-TRAM between January 2018 and August 2021 were included. Female patients aged > 19 years, with breast cancer stage I ~ IIIA (AJCC 8th edition staging), and undergoing immediate breast reconstruction after nipple-sparing, skin-sparing, or total mastectomy or receiving delayed breast reconstruction were enrolled in the study. Patients with a pre-existing hematologic disorder or severe anemia requiring preoperative IV-FCM were excluded from the study. In addition, patients undergoing bilateral breast reconstructions or refusing blood transfusion were excluded.

### Intravenous ferric carboxymaltose (Ferinject)

A total of 84 patients undergoing free MS-TRAM flap breast reconstruction were injected with IV-FCM (Ferinject, Vifor International, Switzerland) from March 2020 to August 2021. A single dose of 500 mg FCM mixed with 100 mL of 0.9% sodium chloride was injected intravenously within five hours from the end of the operation, regardless of the patient's postoperative Hb level or EBL during the surgery. This protocol was adapted from previous protocols on short-term perioperative iron supplementation^25^. The injection was performed slowly over 10 min, and the patients were closely monitored for any adverse reactions.

### Historical control group

The abovementioned IV-FCM injection protocol was adopted at our department in March 2020. Since the adoption, all patients were enrolled as a part of our study cohort (Ferinject group). For comparison, a historical control group of patients undergoing free MS-TRAM breast reconstruction from 2018 to February 2020 was enrolled.

### Surgical procedure

All enrolled patients underwent either immediate or delayed breast reconstruction using a free MS-TRAM flap. During the study period, the surgeons involved were unchanged and surgical techniques or protocols were also not modified. For immediate reconstruction patients, reconstruction was performed following mastectomy and axillary lymph node dissection, if necessary, by a general surgeon. The operation was performed by a plastic surgeon only for delayed reconstruction patients.

In all patients, free MS-TRAM flaps were elevated in either muscle-sparing type I or II while incorporating an average of three to four perforators from medial and lateral rows of the deep inferior epigastric artery. For the recipient vessel, either internal mammary or thoracodorsal vessels were chosen. One arterial and one or two venous anastomoses were performed end-to-end.

### Clinical variables

Preoperative workups, including complete hematological analysis, were performed within one month of the scheduled operation. Hematological parameters including Hb level (grams/deciliter), Hct level (%), RBC count (million count/microliter) were the main clinical variables collected in this study. Other patient characteristics, including BMI, underlying comorbidities, baseline characteristics, along with surgical variables including mastectomy resection amount (grams), execution of axillary lymph node dissection, were collected. Furthermore, any IV-FCM injection-related complications such as hypersensitivity, skin discoloration, and surgical complications (hematoma, wound infection, flap congestion, and necrosis) were analyzed.

### Transfusion

Patients received transfusions according to our strict transfusion protocol. One pack (300 mL) of whole RBC was transfused if the perioperative Hb level was < 8.75 g/dL or the Hct level was < 27%, following the hemoglobin threshold suggested by Kim et al.^8^ If laboratory results following the initial transfusion did not meet the above criteria, additional transfusions were performed until necessary.

### Outcomes

The primary outcomes were transfusion rate and the amount of transfusion (total number of packed RBCs transfused during the hospital stay). The secondary outcomes were fluctuations in hematological parameters and flap or donor site-related complications between the study and control groups. Hematological parameter changes (RBC cell count, Hb level, and Hct level) were analyzed from preoperative levels to immediate postoperative, one-day, two-day, and three-day postoperative levels. Furthermore, any flap or donor site-related complications (re-admission or re-operation within 60 days postoperatively, infection, wound dehiscence, or fat necrosis of the flap or the abdominal donor site) were analyzed.

### Statistical analysis

Baseline characteristics and transfusion rates were compared between the study and historical control groups using univariate analysis. Chi-square tests were performed for binary variables. To reduce the bias of confounding variables between the two groups, propensity score-matched analyses were performed. Logistic regression analysis was utilized to develop propensity scores by incorporating binary responses between the two groups as the dependent variable and IV-FCM injection status and other covariates (age, BMI, surgical risk factors, and operative details) as the independent variables. The propensity score was matched randomly with a 1:2 ratio (study vs. control) with a caliper width of 0.01.

Transfusion frequency and total transfusion amount were compared between the two propensity-matched groups. Subsequently, cross-analysis was performed using the chi-square and Fisher's exact tests. The patients were further grouped into two subgroups within each group depending on whether they received transfusions. Within the subgroups, the primary outcomes (Hb and Hct level changes between preoperative and immediately postoperative and 24 h, 48 h, and 72 h postoperatively) were compared using an independent t-test and Leven's test. A two-sided significance level of 0.05 was set for statistical significance.

## Data Availability

The datasets generated during and/or analysed during the current study are available from the corresponding author on reasonable request.
